# Spatial Variability of the Topsoil Organic Carbon in the Moso Bamboo Forests of Southern China in Association with Soil Properties

**DOI:** 10.1371/journal.pone.0119175

**Published:** 2015-03-19

**Authors:** Houxi Zhang, Shunyao Zhuang, Haiyan Qian, Feng Wang, Haibao Ji

**Affiliations:** 1 State Key Laboratory of Soil and Sustainable Agriculture, Institute of Soil Science, Chinese Academy of Sciences, Nanjing, Jiangsu Province, China; 2 University of Chinese Academy of Sciences, Beijing, China

## Abstract

Understanding the spatial variability of soil organic carbon (SOC) must be enhanced to improve sampling design and to develop soil management strategies in terrestrial ecosystems. Moso bamboo (*Phyllostachys pubescens* Mazel ex Houz.) forests have a high SOC storage potential; however, they also vary significantly spatially. This study investigated the spatial variability of SOC (0-20 cm) in association with other soil properties and with spatial variables in the Moso bamboo forests of Jian’ou City, which is a typical bamboo hometown in China. 209 soil samples were collected from Moso bamboo stands and then analyzed for SOC, bulk density (BD), pH, cation exchange capacity (CEC), and gravel content (GC) based on spatial distribution. The spatial variability of SOC was then examined using geostatistics. A Kriging map was produced through ordinary interpolation and required sample numbers were calculated by classical and Kriging methods. An aggregated boosted tree (ABT) analysis was also conducted. A semivariogram analysis indicated that ln(SOC) was best fitted with an exponential model and that it exhibited moderate spatial dependence, with a nugget/sill ratio of 0.462. SOC was significantly and linearly correlated with BD (*r* = −0.373**), pH (*r* = −0.429**), GC (*r* = −0.163*), CEC (*r* = 0.263**), and elevation (*r* = 0.192**). Moreover, the Kriging method requires fewer samples than the classical method given an expected standard error level as per a variance analysis. ABT analysis indicated that the physicochemical variables of soil affected SOC variation more significantly than spatial variables did, thus suggesting that the SOC in Moso bamboo forests can be strongly influenced by management practices. Thus, this study provides valuable information in relation to sampling strategy and insight into the potential of adjustments in agronomic measure, such as in fertilization for Moso bamboo production.

## Introduction

Soil organic carbon (SOC) is significant in the maintenance of soil fertility and in the dynamics of greenhouse gases because it is a large C pool and plays a potential role as a sink or a source of atmospheric CO_2_ [1–3]. However, SOC varies spatially (in lateral direction) at various scales and in all landscapes [4, 5]. This spatial heterogeneity is related to the variations in numerous factors, including the physicochemical properties of soil, topography, climate, parent material, land use patterns, and management practices [6, 7]. The correlations between SOC and either soil properties or spatial variables are complex. As a result of these characteristics, the relevant processes and mechanisms are difficult to predict [8]. Thus, the understanding regarding SOC spatial variation must be enhanced by inclusion of soil properties and spatial variables to improve SOC sampling design, to develop soil management strategies, and to assess the role of SOC in mitigating global climate warming [7]. Previous studies have shown that SOC variability can be investigated effectively through geostatistics [4, 6]. Classical statistics was considered to be unsuitable for describing spatial dependency due to its complete assumption of independent measurements [4]. However, geostatistics takes into account both the structured and random characteristics of soil observations in data processing through a set of statistical tools. Thus, spatial patterns can be described and modeled, un-sampled locations predicted, and the uncertainty attached to these predictions assessed [4].

Moso bamboo (*Phyllostachys pubescens* Mazel ex Houz.) is the primary bamboo type in China and covers an area of approximately 3.0 × 10^6^ ha. This area accounts for 71.9% of the total bamboo area in this country. Moso bamboo grows naturally in subtropical monsoon climate zone (in summer high temperatures and rainy; in winter cold and dry). It grows at elevations between 10 to 1700 meters above sea level but most of the area is less than 800 m and in the hills and mountains [9]. It takes about two months for the shoots to emerge and grow into new culms. Moso bamboo forests are renewable and versatile. Moreover, they possess both ecological and economic value. This forest area continues to increase at an annual rate of approximately 3%, suggesting that Moso bamboo forest may have constantly increasing carbon storage in China [10]. SOC (0 to 60 cm in depth) storage accounts for approximately 66.7% of the C storage in Moso bamboo forests [11]. Thus, the distribution of SOC in the organic–mineral complex has often been analyzed recently, along with the temporal dynamics of SOC and the effects of fertilization management on SOC [10, 12–15]. However, understanding regarding SOC variability remains vague, as is that related to the influences of environmental factors on the spatial variation of SOC in Chinese Moso bamboo forests. Over 60% of the rhizomes of Moso bamboo grow horizontal and expand within the surface layer of 20 cm in depth [12]. In addition, the dead vegetation and bamboo leaves would return to the topsoil (0–20 cm) and the decomposition of them usually occurs in the topsoil. Therefore, in our current research, we studied spatial variability of SOC in the topsoil which was affected most by the environment and would better represent the variability of SOC in Moso bamboo forest.

The current study is conducted on the Moso bamboo forests in Jian’ou City (county-level city), southern China, and it aims (1) to map SOC spatial distribution using geostatistics and geographic information system (GIS) facilities and to assess the characteristics of its spatial distribution patterns; (2) to quantitatively determine the complex relationships between environmental variances and SOC; and (3) to provide background for decisions regarding sampling design and forestry management.

## Materials and Methods

### Study area description

Jian’ou City (117°58′ to 118°57′ E, 26°38′ to 27°21′ N) is located in the northern part of Fujian Province, southern China. It has a total area of 4214.0 km^2^ and lies at the center of the Moso bamboo distribution in China [16]. At 8.63 × 10^4^ ha, this city also houses the largest Moso bamboo area in China at the county scale [17]. The mountain, hill, and valley basin regions account for 57%, 30%, and 13% of the total area of the county, respectively. The study area is characterized by a subtropical marine monsoon climate. The mean annual temperature is approximately 17–21°C, and average annual precipitation is 1600–1800 mm. Sixty percent of mean annual precipitation occurs in the rainy season (from March to June), whereas only 21% occurs during the dry season (from October to the following February). The sunny season lasts for 1842 h annually, and the duration of the frost-free period is approximately 280–290 d. The major soil types (subgroup) in this county are red soil, yellow soil, lateritic red soil and yellowish red soil according to the soil genetic classification of China, and the parent materials of soil primarily include red sandstone, alluvium, Quaternary red clay, and shale [18]. The understory plants in Moso bamboo forests in this area include *Digitaria sanguinalis*, *Paederia scandens*, *Houttuynia cordata*, *Ampelopsis aconitifolia*, *Cyperus diffomis*, *Dicranopteris linearis*.

### Soil sampling and analysis

**Fig 1:**
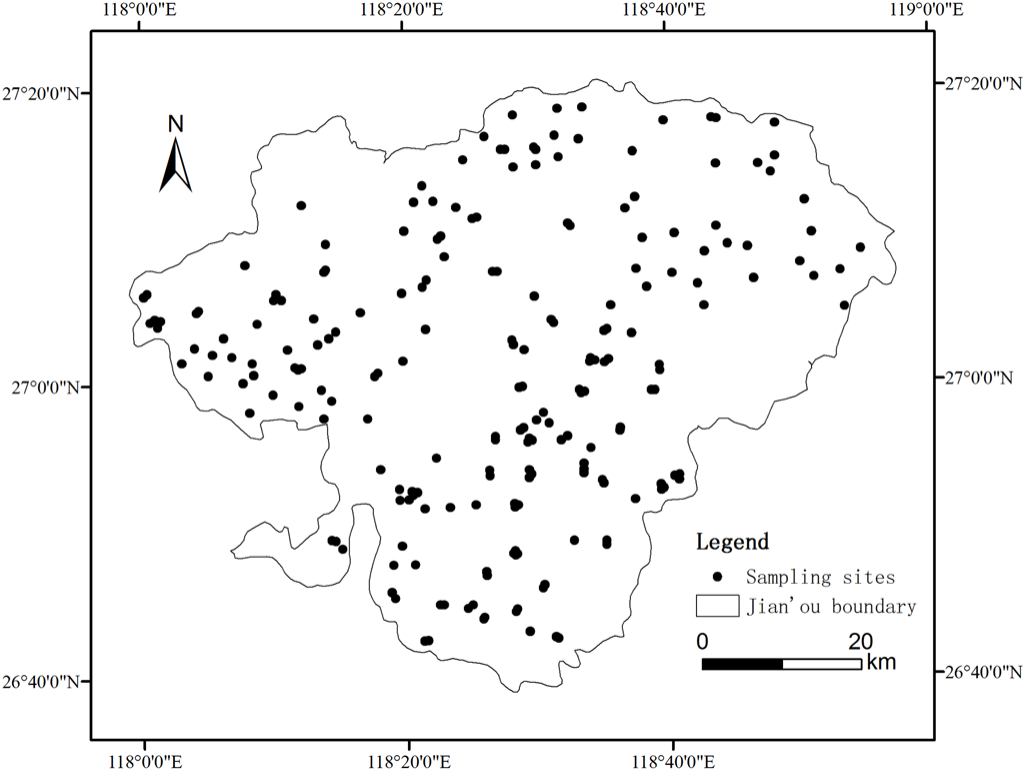
Spatial distribution of soil samples in Jian’ou City, southern China.

209 soil samples (0 cm to 20 cm) were collected in August 2009. The sampling locations were homogeneously distributed in Jian’ou City, and permission to enter each location was given by Fujian Jiou’ou Forestry Bureau, China. The coordinates of the locations were recorded by a global positioning system and the distribution of sampling sites is presented in Fig. 1. We obtained five soil samples at random from each sampling location within a radius of approximately 50 cm using an auger with a diameter of 5 cm. The five samples were then mixed to obtain the representative soil sample of the sampling point. All samples were air-dried, ground to pass through 2 mm sieves, and stored for further analysis. Another soil core samples were collected from each sampling location (0–20 cm) for bulk density (BD) determination.

There was no need of approval by Institutional Review Board (IRB) or Ethics Committee or by an Institutional Animal Care and Use Committee (IACUC) or equivalent animal ethics committee because our study was not human subject research and our object was Moso bamboo forest which was a plantation but not an animal.

Soil BD was determined using the core method, gravel content (GC) was obtained using the weight method, and soil pH was determined through potentiometry [19]. Soil organic matter (SOM) was obtained with the potassium-dichromate external heating method [20]. SOC was then derived from SOM using the van Bemmelen coefficient (1.724). Soil cation exchange capacity (CEC) was determined by ammonium acetate extraction buffered at pH 7 [19].

### Statistical and geostatistical methods

In this study, the central trend and the spread of data were described by the following statistical parameters: the mean, median, standard deviation, coefficients of variation, maximum and minimum values, and the skewness and kurtosis of the dataset. The normal frequency distribution of data was verified by the Kolmogorov–Smirnov (K–S) test.

The semivariogram of geostatistics [21, 22] was used to measure the spatial variability of a regionalized variable and to generate the input parameters for the Kriging method of spatial interpolation. The semivariogram is half of the expected squared difference between paired data values *Z (x)* and *Z (x + h)* to the lag distance *h* by which locations are separated [21]. For discrete sampling sites such as those in this study, the function is usually written in the following form:

$$\gamma \left(h\right)=\frac{1}{2N\left(h\right)}\sum_{i=1}^{N\left(h\right)}{\left[Z\left(x_{i}\right)-Z\left(x_{i}+h\right)\right]}^{2},$$ (1)

where *Z (x*_*i*_ *)* is the value of the variable *Z* at location *x*_*i*_; *h* is the lag; and *N (h)* denotes the number of pairs of sampling points separated by *h*. The distance between the sample pairs is rarely equal to *h* in irregular sampling. That is, *h* is often represented by a distance interval.

Experimental variograms were generated by calculating the variogram at different lags. Spherical, exponential and Gaussian models were selected to fit these variograms, as well as to investigate the spatial structure and the input parameters for Kriging interpolation. The best-fitting model for the variable would be chose based on the determination coefficients (R^2^).

The spherical model is given by

$$\gamma (h)=\{\begin{array}{c} C_{0}+C\left[\frac{3}{2}\frac{h}{a}-\frac{1}{2}\frac{h^{3}}{a^{3}}\right]\;0<h\leq a\text{​​​​ }\\ C_{0}+C\;h>a \end{array},$$ (2)

The exponential model is given by

$$\gamma \left(h\right)=C_{0}+C\left[1-\exp\left(-\frac{h}{a}\right)\right],$$ (3)

The Gaussian model is given by

$$\gamma (h)=C_{0}+C\left[1-\exp(-\frac{h^{2}}{a^{2}})\right],$$ (4)

where *C*_*0*_ is the nugget variance *(h = 0)*that represents the experimental error and field variation within the minimum sampling space. The variogram increases with the increase in lag distance to either attain or approach a maximum value or sill *(C*_*0*_ *+ C)* that is almost equivalent to the population variance, i.e., a priori variance. *C* is the structural variance, whereas *a* is the spatial range across which the data are correlated spatially.

Validation of the best-fitting models before carrying out spatial prediction (interpolation) is an important step to ensure model quality. Assessment of the best-fitting model quality was performed using leave-one-out cross-validation (LOOCV) where each observation was removed from the data set and the SOC at that location was predicted using the remaining observations. Two measures of model quality were calculated:

Mean error (ME):

$$\text{ME}=\frac{1}{\text{n}}\sum_{i=1}^{\text{n}}\left({\text{Z}}_{\text{i}}-{\overset{\wedge }{\text{Z}}}_{\text{i}}\right),$$ (5)

Root mean square error (RMSE):

$$\text{RMSE}=\sqrt{\frac{1}{\text{n}}\overset{\text{n}}{\underset{\text{i}=1}{\sum }}{\left({\text{Z}}_{\text{i}}-{\overset{\wedge }{\text{Z}}}_{\text{i}}\right)}^{2}},$$ (6)

Where, Z_i_ is the measured SOC while ${\overset{\wedge }{\text{Z}}}_{\text{i}}$ is the predicted SOC from LOOCV. A good model has a value close to zero for ME and RMSE.

In this study, a traditional statistical analysis was conducted using SPSS 17.0 (SPSS Inc., Chicago, IL, USA). The geostatistical analysis utilized GS+ 9.0 software (Gamma Design Software LLC, Plainwell, MI). Spatial interpolation maps were produced using the GIS software ArcGIS 9.3 (ESRI, Redlands, CA) based on the variogram parameters calculated using the GS+ software.

### Topography

**Fig 2:**
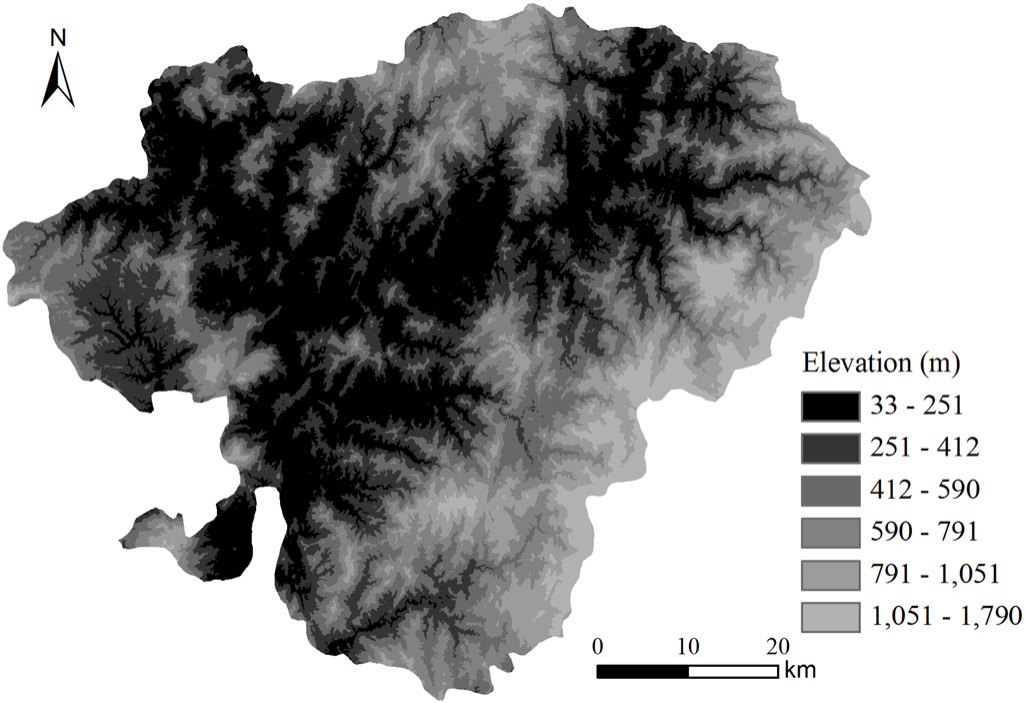
DEM of Jian’ou City, southern China.

The soil sampling process did not provide topographic information (spatial variables), such as elevation, slope, and aspect (expressed in positive degrees from 0 to 359.9 and measured clockwise from north). Thus, a regular 30 m grid digital elevation model (DEM) was used to derive the elevation, slope, and aspect data for each soil sampling point. The DEM was based on the recordings of the Advanced Spaceborne Thermal Emission and Reflection Radiometer (ASTER) as provided by the National Aeronautics and Space Administration (NASA) and the Japanese Ministry of Economy, Trade and Industry (METI) in 2009. The digital elevation maps of Jian’ou are shown in Fig. 2. The slope and aspect map were processed based on the DEM using ArcGIS 9.3. The elevation, slope, and aspect data of each soil sampling point were derived from the DEM according to the sample positions recorded by GPS. The linear correlation between SOC and the spatial variables (elevation, slope, and aspect) was analyzed using SPSS 17.0.

### Aggregated boosted tree (ABT) analysis

Traditional statistical models such as linear regression are routinely used to explain relationships among data relationships in simple terms. As a result, they fail to quantify the complex interactions between the variances and responses [23]. However, ABT which is a statistical learning method that aims to attain both accurate prediction and explanation can quantitatively determine these complex relationships, including nonlinearities. Therefore, it can explain underlying processes [23]. In addition, ABT, based on BTs, can deal with many types of response variables (numeric, categorical and censored) and environmental variables (numeric, categorical) and has been widely applied in ecological studies [23, 24].

In this study, ABT analysis was conducted using the gbmplus package in R language 2.7.1 and was used to evaluate the relative influence of four physicochemical variables (GC, CEC, BD, and pH) and three spatial variables (slope, elevation, and aspect) on SOC. The variance importance figure was plotted using Matlab 7.0 (MathWorks Inc., Natick, MA, USA).

### Estimation of variance

Cline [25] summarized the sampling principles followed by soil scientists and provided the classical formulas used to estimate the means and variances and to determine the number of observations (sample size) that generates the desired estimation variance. Thus, if the true mean is *μ* and a deviation of *x—μ* is acceptable in its estimation, then the required sample size *n* can be calculated as follows:

$$n=t_{\alpha }^{2}S^{2}/{\left(x-\mu \right)}^{2},$$ (7)

where *S* ^*2*^ is the estimated variance and *tα* is the value of Student’s *t* at the chosen level of probability *α*.

The ignorance of global variability may increase costs unnecessarily in sample collection and analysis, whereas the neglect of local variability can aggravate estimation errors or uncertainty [26]. An enhanced appreciation of the spatial variability of soil properties may improve the applied sampling strategy [27]. McBratney and Webster [27] also proposed equations to calculate standard error (SE) based on the semivariance of a given dataset using Kriging (Equations 8 and 9) in consideration of the spatial dependency of soil properties. Nonetheless, the Thiessen polygon should be constructed prior to calculation. *S* is a triangular grid in this polygon; the observation points at its center and at the side are equal to the sampling interval. The estimation variance of its average value is expressed as

$$\sigma_{S}^{2}=2\bar{\gamma }\left(x,S\right)-\bar{\gamma }\left(S,S\right),$$ (8)

where *n* is the number of samples required to estimate the mean value; $\bar{\gamma }\left(x,S\right)$is the average semivariance between the central point, **x**, and all other points in the grid; and $\bar{\gamma }\left(S,S\right)$ is the variance within the grid.

Furthermore, SE is calculated as:

$$\text{SE}\approx \sqrt{\frac{1}{\text{n}}\sigma_{\text{S}}^{2}},$$ (9)

ArcGIS 9.3 is used to construct the Thiessen polygon based on the sampling points. In this study, the SE figure was plotted using Matlab 7.0.

## Results

### Descriptive statistics

Table 1 summarizes the descriptive statistics for the soil physicochemical parameters of the 209 soil samples obtained from the Moso bamboo forests in Jian’ou City. All of the soil properties were similar in terms of mean and median values, thereby indicating that the dataset of the soil properties was evenly and normally distributed. Soil pH ranged from 3.85 to 6.02, GC ranged from 1.10% to 60.40%, BD ranged from 0.76 g cm^−3^ to 1.19 g cm^−3^, and SOC ranged from 0.42% to 6.48%.

The coefficient of variation (CV) is a major indicator of the variability of soil properties. A variable is considered weakly variable when the CV is less than 10%. A variable is moderately variable when the CV is between 10% and 100%. Otherwise, a variable is strongly variable [28]. Except for pH (CV = 6.42%), the soil variability data in Table 1 indicated that BD had the lowest CV (*CV* = 8.05%), suggesting a weak variability. The CVs for SOC, GC, and CEC ranged from 12.08% to 96.40%, thereby indicating a moderate variability.

**Table 1:** Descriptive statistics of soil variables^a^.

Variable	Mean	Median	SD	CV (%)	Skewness	Kurtosis
BD (g cm^−3^)	0.95	0.95	0.08	8.44	0.42	0.36
GC (%)	18.71	17.20	10.62	56.76	1.17	2.06
pH	4.94	4.96	0.32	6.42	0.07	0.69
CEC (mmol_+_ kg^−1^)	39.17	3.87	4.73	12.08	4.17	38.85
SOC (%)	2.37	2.02	1.12	47.42	1.31	1.96

^a^SD = standard deviation, CV = coefficient of variation, BD = bulk density, GC = gravel content, CEC = cation exchange capacity and SOC = soil organic carbon

A normal distribution is desirable for a studied variable in linear geostatistics and in conventional statistics [29]. Serious deviations from normality, such as excessive skewness, can impair the variogram structure and the Kriging results. The quantitative parameters of the probability distribution and the significance level of the K–S test were thus calculated for conformance to a normal distribution. The results indicated that the SOC data passed the K–S normality test at a significance level of 0.05 after logarithmic transformation.

Table 2 displays the linear correlation coefficients among the five variables. SOC was significantly correlated with BD (*r* = −0.373**), pH (*r* = −0.429**), GC (*r* = −0.163*), and CEC (*r* = 0.263**). Variables such as BD and GC (*r* = 0.451**), pH and BD (*r* = 0.206**), and pH and GC (*r* = 0.353**) were also significantly correlated as generally reported.

**Table 2:** Correlation coefficients among selected soil properties of the Moso bamboo forest in Jian’ou City, southern China.

Variable^a^	BD	GC	pH	CEC
GC	0.451**			
pH	0.206**	0.353**		
CEC	−0.082	−0.093	−0.028	
SOC	−0.373**	−0.163*	−0.429**	0.263**

*,**Significant at P = 0.05 and P = 0.01 levels, respectively

^a^The number of SOC observations was 209. BD = bulk density, GC = gravel content, CEC = cation exchange capacity and SOC = soil organic carbon

### Geostatistical analysis

**Fig 3:**
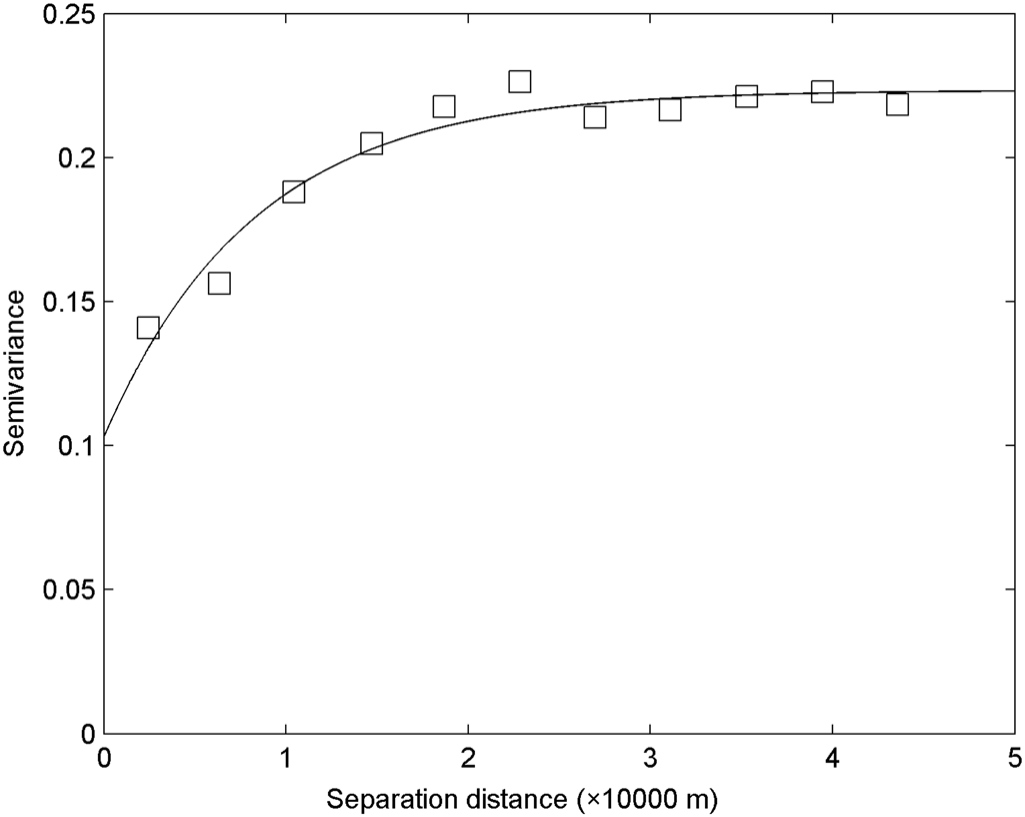
Experimental semivariograms with the best-fit model (exponential) for SOC [ln(SOC)]

Geostatistics has mainly been used to estimate and map soil chemical properties in un-sampled areas through semivariogram analysis [7]. The parameters of the four models fitted to the SOC semivariogram are shown in Table 3. The best-fitting model for ln(SOC) was selected based on the determination coefficients (*R* ^2^). A model with the maximum R^2^ was preferable; therefore, ln(SOC) was best fitted with an exponential model (*R* ^2^ = 0.953). The determination coefficients of ln(SOC) were greater than 0.9, thereby indicating that these measured parameters can be modeled with a high degree of confidence. Fig. 3 presents the semivariogram and best fitted model (exponential) for ln(SOC).

**Table 3:** Semivariogram models and model parameters for ln(SOC) in the Moso bamboo forest of Jian’ou City, southern China

Model	Nugget	Sill	Nugget/sill ratio	Range (m)	*R* ^2^
Linear	0.1635	0.2375	0.688	42586.11	0.652
Spherical	0.0002	0.2084	0.001	15996.88	0.924
Exponential	0.1032	0.2234	0.462	24870.00	0.953
Gaussian	0.0257	0.2084	0.123	11750.00	0.867

### Kriging map for SOC

**Fig 4:**
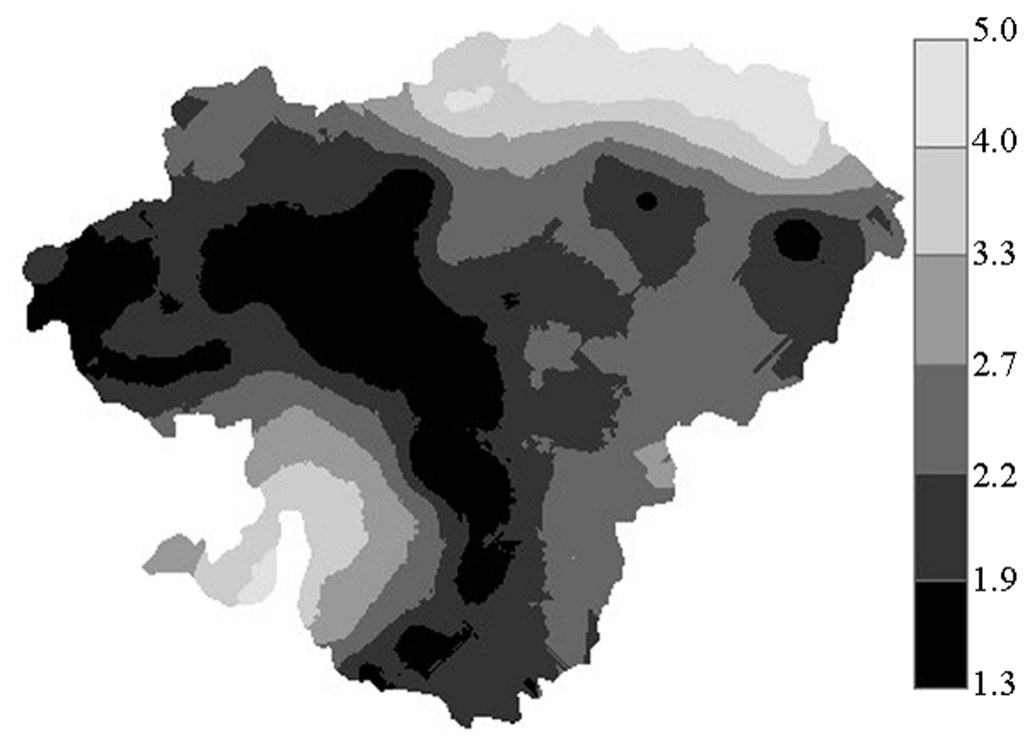
Spatial distribution of SOC (%) interpolated by ordinary Kriging for Moso bamboo stands in Jian’ou City, southern China

The LOOCV result showed that ME and RMSE for spatial interpolation of SOC were 0.011 and 0.852, respectively. The RMSE provides a measure of interpolation precision, with lower values indicating more precise methods, while the ME measures the bias (Gumiere et al. 2014). The ME values close to zero indicated that the selected model was unbiased. The two criteria for interpolation of SOC indicated that the interpolation method performed well based on the parameters from the best-fitting model for ln(SOC). Fig. 4 showed the interpolation map of the SOC of the study area produced by ordinary Kriging. Although the Kriging map covers all soils, including non-soils, the non-Moso bamboo forest soil blocks in the map do not represent the actual values because we collected data only in relation to Moso bamboo forest soil. That is, the map values can only be applied to Moso bamboo forest soil.

Kriging maps represent the detailed spatial distribution of SOC. Based on the interpolation map of SOC contents, SOC concentrations were lowest in the center of the study area, which extends to the northwest and the southwest. By contrast, SOC content increases at the edge of the city, such as in the north and southeast areas. The statistical result shows that the area with the lowest SOC contents, which range between 1.3% and 1.9%, accounted for 18.43% of Jian’ou City. The area with the highest values, which range from 4.0% to 5.0%, constituted 6.31%.

### Analysis of the correlation between topographical factors and SOC

The linear correlation between SOC and topographic factors was performed. SOC was significantly and positively correlated with elevation in the investigated surface layer (0 cm to 20 cm) of the soil in the Moso bamboo forests of Jian’ou City (*r* = 0.192, P < 0.05). However, SOC and slope degree (aspect) were not significantly correlated in Moso bamboo forests.

### ABT analysis of the contributions of variables to SOC variability

**Fig 5:**
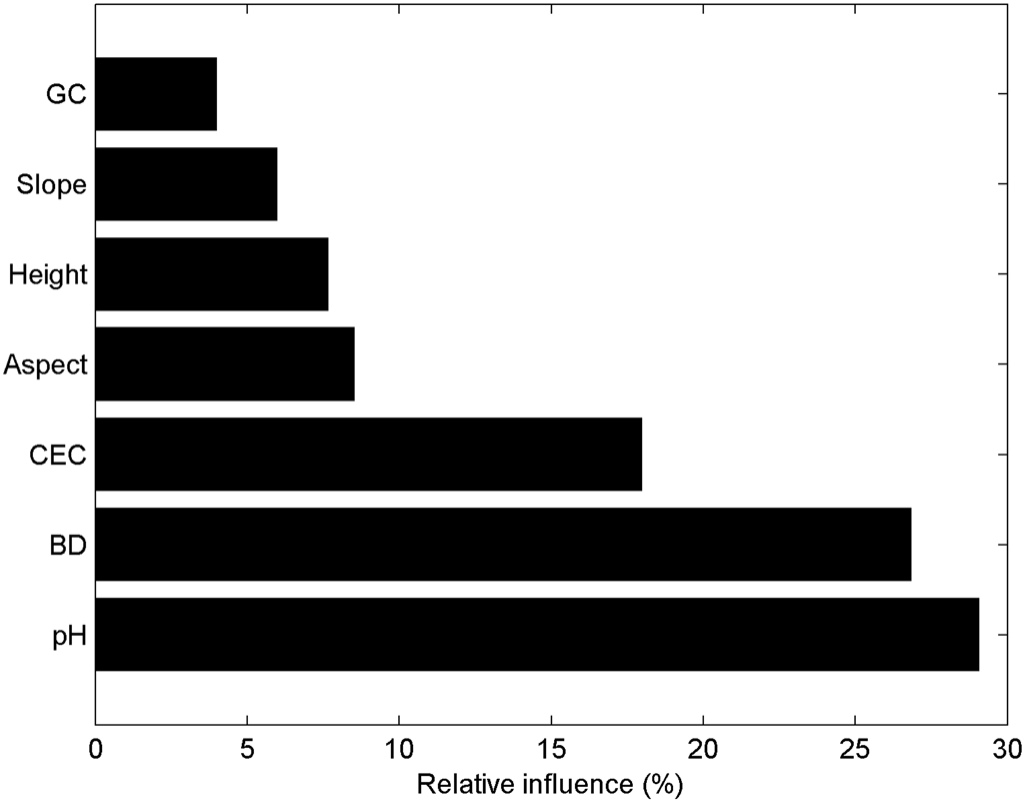
Contribution percentages of the four physicochemical variables (GC, CEC, BD, and pH) and of the three spatial variables (slope, elevation, and aspect) to SOC variation as revealed by ABT analysis

The ABT analysis results suggested that the contribution percentages of the seven variables (pH, BD, CEC, aspect, elevation, slope, and GC) to SOC variation vary significantly from 4.00% to 29.06% (Fig. 5). The variable importance figure indicated that the four physicochemical variables (pH, BD, GC, and CEC) accounted for 77.86% of SOC variation, whereas the three spatial variables (aspect, elevation, and slope) accounted for 22.14%. Of the three physicochemical variables, pH had the highest contribution percentage to SOC variation, followed by BD. Among the three spatial variables, aspect had the highest contribution percentage, followed by elevation.

### Estimation of SE using two methods

**Fig 6:**
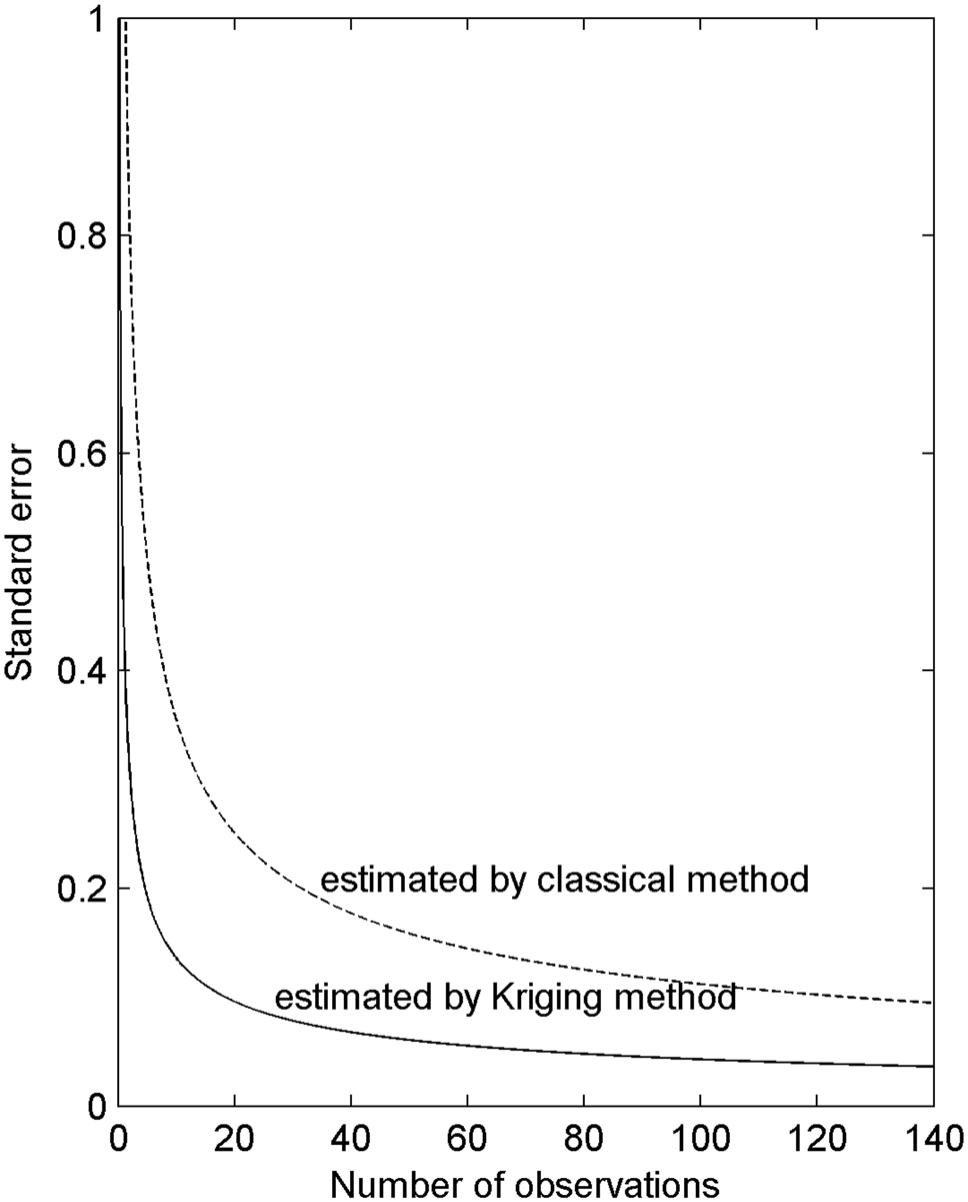
SEs of SOC as estimated using the classical and Kriging methods for Moso bamboo stands in Jian’ou City, southern China

The SOC estimation variances and their square roots (the SEs) were calculated using the classical (Equation 7) and Kriging methods (Equations 8 and 9) for the SOC in the soil of Moso bamboo forests. The results obtained with these methods are plotted as SE versus sample size (Fig. 6). In Fig. 6, the SEs (estimated) calculated using the classical and Kriging methods decreased with the increase in the number of observations to a maximum of 40. Beyond this limit, the trend leveled off.

## Discussion

### Relationship between SOC and soil properties

SOC is an indicator of soil productivity [6]. However, it is a highly spatial variable because it is affected by weather, soil texture, soil porosity, vegetation, and topography [30, 31]. SOC content was significantly and negatively correlated with pH in the investigated surface layer (0 cm to 20 cm) of the Moso bamboo forest. This result indicated that SOC content decreased with the increase in pH, which is consistent with the results of other studies. These studies indicate that soil pH affected the regulation of the decomposition of fresh organic matter considerably along with SOM decomposition by influencing microbial activity [31–34]. Shi et al. (2012) also observed a negative relationship between SOC and soil pH and concluded that acidification inhibits SOC decomposition; thus, general C loss from soils is insignificant. However, our finding differs from that of Weigand et al. [35] who did not observe a correlation between SOC and soil pH. This discrepancy may be attributed to their use of various soils derived from different parent materials under varied climatic conditions.

The significant and negative correlation between SOC and BD was confirmed by other studies [31, 36, 37]. The reduction in BD can be attributed to the increased organic matter content in the soil, the enhanced aggregation and consequent increase in the volume of micropores [38], and the increased root growth of Moso bamboo. Furthermore, SOC and CEC were significantly and positively correlated in accordance with the results reported by other studies [6, 39].

The variable GC is generally neglected in previous studies of SOC variation in forest soil. However, the Moso bamboo usually grows on mountains with much gravel. Moreover, SOC was significantly and negatively correlated to GC, as per the current study. Olaleye et al. [39] reported a similar result. The increased contents of subsoil gravel reduce soil porosity and available water-holding capacity, and they increase soil compaction. As a result, root growth is inhibited [39, 40]. Therefore, the GC is a variable that is essential to the study of SOC variability in Moso bamboo forests.

### Geostatistical analysis

In geostatistics, the range of the semivariogram is the maximum distance over which the soil properties of two samples are related. Thus, it can be an effective criterion for the evaluation of sampling design and the mapping of soil properties [41, 42]. Table 3 shows that the spatial correlation (range) of ln(SOC) was 24870 m. No spatial dependence (autocorrelation) was observed in the soil properties when the separation distance between two samples was out of range. By contrast, soil properties are similar (spatial correlation) when the distance is short and is within the range. Therefore, the sampled points cannot be used for either interpolation or extrapolation when the distance between the sampled and the predicted points is longer than the model range. The observed values of the soil properties in a wide range are influenced by other values of these properties over longer distances than soil properties with smaller ranges, according to Zhang et al. [41]. Thus, the ln(SOC) range of approximately 24870 m in the study area indicates that ln(SOC) values influenced the neighboring ln(SOC) values over longer distances than other soil variables with a smaller range.

The nugget/sill ratio can generally be used to classify the spatial dependence of soil properties [43]. A variable displays strong spatial dependence if the ratio is less than 0.25 and moderate spatial dependence if the ratio lies between 0.25 and 0.75. Otherwise, the variable has weak spatial dependence. In the study area, ln(SOC) with nugget/sill ratios of 0.50 showed moderate spatial dependence. This result may be attributed to the comparable effect of intrinsic (soil-forming processes) and extrinsic (soil fertilization and cultivation practices) factors [44].

### Spatial distribution of SOC

The block distribution (not patchy distribution) in the SOC spatial distribution maps suggests that SOC displayed moderate spatial dependence. This finding is consistent with the result of the geostatistical analysis, which indicates that SOC attained mid-range values (Table 3). This result also suggests that SOC was affected by both the natural environment and human activities such that its spatial distribution did not display a significant geographic trend.

The spatial distribution over the entire region could be clearly derived from the Kriging SOC maps. Therefore, the Moso bamboo forests in Jian’ou City may be classified into groups based on similar SOC contents. Different groups can be subject to appropriate fertilization to manage soil precisely and efficiently [7]. Moreover, the Kriging SOC maps can be used to accurately estimate C storage in Moso bamboo forests. In the process, we can precisely assess the role of Moso bamboo in the mitigation of global warming.

### Effect of topographical factors on SOC

SOC was significantly and positively correlated with elevation. This finding is consistent with the results of other studies [2, 45]. Previous studies demonstrated that temperature decreases with an increase in elevation and that this relationship is a key factor that controls the rate of organic matter decomposition [46, 47]. An increase in temperature enhances soil respiration rate and increases the chances of C loss from the soil to the atmosphere as CO_2_. The area with a low SOC value is located at the center of the region, according to the spatial interpolation map (Fig. 4).

### Contribution of variables on SOC variation

The sum of the contribution percentages of the physicochemical variables to SOC variation is larger than that of the contribution percentages of the spatial variables, which indicated that the physicochemical variables of soil affected SOC variation more significantly than spatial variables. Although spatial variables significantly influenced SOC in previous studies [48–50], they also controlled the hydrothermal regime (including temperature, precipitation and soil moisture), which in turn influenced soil properties. Accordingly, the values of the spatial variables were high and indirectly affected SOC. Therefore, we expect soil physicochemical variables to influence SOC variation more strongly than spatial variables do.

According to the Pearson correlation analysis, elevation was significantly and positively correlated with SOC, whereas there was no significant correlation between aspect and SOC. However, the ABT analysis revealed that aspect has a higher contribution percentage to SOC variation than elevation. This finding may be attributed to the fact that the correlation of aspect with SOC is more nonlinear than that of elevation with SOC. As mentioned above, the Pearson correlation analysis can determine only the linear correlation between SOC and spatial variables, whereas the ABT analysis can also detect the nonlinear correlation between SOC and spatial variables.

### SOC sampling strategy

Fig. 6 shows that the advantage of Kriging estimation increases with the increase in sample size or specifically, sampling intensity. This advantage holds in all instances in which spatial dependence is observed. The extent to which the advantages diminish with sparse sampling depends on the semivariogram. Kriging estimates are more precise than those derived using classical methods given 10 or more samples because the semivariogram of the SOC was exponential and displayed a moderate range, although its nugget variance was fairly small. The sampling interval exceeds the range of the semivariogram with few samples. Therefore, the two variance estimates converge.

The results shown in Fig. 6 can be used to determine the number of samples to be collected given an expected SE level. The investigator decides the tolerable level of error and determines the corresponding sample size from the lower of the two curves. For instance, the number of SOC samples to be obtained from the study area should be approximately 10 on the grid using the Kriging method and approximately 64 on the grid using the classical method if the allowable SE is 0.15. The advantage of the Kriging estimate is enhanced with a small required SE. Thus, the sample size should be approximately 19 using the Kriging method and 125 when the classical method is utilized for an expected SE of 0.1. Therefore, the classical method is more challenging to apply than the Kriging method.

## Conclusions

SOC varies spatially at various scales in all landscapes. However, the correlations between SOC and either soil properties or spatial variables are highly complex. Therefore, this study applied geostatistical, GIS, and ABT approaches to explore the relationship between SOC and either soil properties or spatial variables. The geostatistical analysis showed that ln(SOC) exhibited moderate spatial dependence and that the spatial SOC map could be illustrated through Kriging interpolation. This map can be used to assess soil fertility and to estimate C storage. Moreover, the results of variance analyses that considered both the classical and Kriging methods suggested that the advantages of Kriging estimates are enhanced as sample size increases given an expected SE level. Moreover, the Kriging method required fewer samples than the classical method. An ABT analysis also revealed that the physicochemical variables of soil affected SOC variation more strongly than spatial variables, thereby suggesting that the SOC in Moso bamboo forests can be influenced significantly by management practices. Thus, this study provides valuable information regarding sampling strategy and insight into the potential of adjustments in agronomic measure, such as in fertilization.

## Supporting Information

S1 Table The specific locations of sampling sites (209) recorded by Global Positioning System (GPS). Soil type (subgroup) is based on the genetic soil classification of China.
(DOC)
