# Supplementary material for: Spatial Variability of the Topsoil Organic Carbon in the Moso Bamboo Forests of Southern China in Association with Soil Properties
Source: PLoS One. 2015 Mar 19;10(3):e0119175. doi: 10.1371/journal.pone.0119175 (PMC4366393; doi:10.1371/journal.pone.0119175)
Supplement: S1 Table — Soil type (subgroup) is based on the genetic soil classification of China. (DOC) [file pone.0119175.s001.doc]

**Supplemental information**

**S1 Table The specific locations of sampling sites (209) recorded by Global Positioning System (GPS). Soil type (subgroup) is based on the genetic soil classification of China**

| Sampling site number | Longitude | Latitude | Elevation | Management type | Soil type (subgroup) |
| --- | --- | --- | --- | --- | --- |
| 1 | 118.324774E | 26.881041N | 245 | Extensive | Red soil |
| 2 | 118.371768E | 26.91605N | 214 | Extensive | Red soil |
| 3 | 118.340341E | 26.878253N | 203 | Extensive | Red soil |
| 4 | 118.341403E | 26.873794N | 170 | Extensive | Red soil |
| 5 | 118.421075E | 26.862327N | 413 | Extensive | Red soil |
| 6 | 118.388798E | 26.859991N | 313 | Extensive | Red soil |
| 7 | 118.356236E | 26.858717N | 156 | Extensive | Red soil |
| 8 | 118.318226E | 26.757428N | 249 | Extensive | Red soil |
| 9 | 118.31398E | 26.764223N | 257 | Extensive | Red soil |
| 10 | 118.316528E | 26.795226N | 257 | Extensive | Red soil |
| 11 | 118.344133E | 26.795438N | 397 | Extensive | Red soil |
| 12 | 118.327357E | 26.81646N | 222 | Extensive | Red soil |
| 13 | 118.30075E | 26.903522N | 174 | Extensive | Red soil |
| 14 | 118.346992E | 26.877404N | 206 | Extensive | Red soil |
| 15 | 118.28527E | 26.960926N | 104 | Extensive | Red soil |
| 16 | 118.325022E | 26.868343N | 580 | Extensive | Red soil |
| 17 | 118.336206E | 26.86889N | 503 | Extensive | Red soil |
| 18 | 118.252366E | 26.813941N | 499 | Extensive | Red soil |
| 19 | 118.243491E | 26.822816N | 488 | Extensive | Red soil |
| 20 | 118.238582E | 26.82376N | 948 | Extensive | Red soil |
| 21 | 118.446856E | 26.94N | 132 | Extensive | Red soil |
| 22 | 118.439333E | 26.901546N | 258 | Extensive | Red soil |
| 23 | 118.439611E | 26.895416N | 186 | Extensive | Red soil |
| 24 | 118.446856E | 26.936377N | 151 | Extensive | Red soil |
| 25 | 118.50788E | 26.966471N | 321 | Extensive | Red soil |
| 26 | 118.498685E | 26.958112N | 147 | Extensive | Red soil |
| 27 | 118.514846E | 26.954768N | 211 | Extensive | Red soil |
| 28 | 118.488932E | 26.901546N | 376 | Extensive | Red soil |
| 29 | 118.488932E | 26.892908N | 411 | Extensive | Red soil |
| 30 | 118.492273E | 26.896968N | 381 | Extensive | Red soil |
| 31 | 118.470234E | 26.863602N | 707 | Extensive | Yellowish red soil |
| 32 | 118.47075E | 26.859601N | 765 | Extensive | Yellowish red soil |
| 33 | 118.474493E | 26.862053N | 770 | Extensive | Yellowish red soil |
| 34 | 118.478622E | 26.947094N | 186 | Extensive | Yellowish red soil |
| 35 | 118.482364E | 26.949416N | 197 | Extensive | Yellowish red soil |
| 36 | 118.47759E | 26.995227N | 184 | Extensive | Yellowish red soil |
| 37 | 118.482106E | 26.99626N | 272 | Extensive | Yellowish red soil |
| 38 | 118.487913E | 26.933286N | 374 | Extensive | Red soil |
| 39 | 118.493591E | 26.935351N | 264 | Extensive | Red soil |
| 40 | 118.48972E | 26.937544N | 256 | Extensive | Red soil |
| 41 | 118.673178E | 26.893699N | 1234 | Extensive | Yellow soil |
| 42 | 118.678723E | 26.889667N | 1023 | Extensive | Yellow soil |
| 43 | 118.679731E | 26.895211N | 1030 | Extensive | Yellow soil |
| 44 | 118.5832E | 26.885886N | 573 | Extensive | Red soil |
| 45 | 118.581436E | 26.889667N | 599 | Extensive | Red soil |
| 46 | 118.585972E | 26.816575N | 602 | Extensive | Red soil |
| 47 | 118.586224E | 26.820859N | 700 | Extensive | Red soil |
| 48 | 118.545394E | 26.821616N | 843 | Extensive | Yellowish red soil |
| 49 | 118.604724E | 26.948896N | 504 | Extensive | Red soil |
| 50 | 118.604472E | 26.94562N | 515 | Extensive | Red soil |
| 51 | 118.62277E | 26.867235N | 815 | Extensive | Yellowish red soil |
| 52 | 118.557996E | 26.902017N | 423 | Extensive | Yellowish red soil |
| 53 | 118.557996E | 26.897732N | 530 | Extensive | Red soil |
| 54 | 118.558248E | 26.908318N | 433 | Extensive | Red soil |
| 55 | 118.553736E | 26.992247N | 819 | Extensive | Red soil |
| 56 | 118.567346E | 26.926213N | 475 | Extensive | Red soil |
| 57 | 118.556005E | 26.988467N | 821 | Extensive | Yellowish red soil |
| 58 | 118.560289E | 26.990231N | 210 | Extensive | Yellowish red soil |
| 59 | 118.655561E | 26.884122N | 899 | Extensive | Yellowish red soil |
| 60 | 118.656065E | 26.878325N | 868 | Extensive | Yellowish red soil |
| 61 | 118.354865E | 26.709208N | 1005 | Extensive | Yellow soil |
| 62 | 118.359314E | 26.70955N | 150 | Extensive | Red soil |
| 63 | 118.375045E | 26.749706N | 201 | Extensive | Red soil |
| 64 | 118.379152E | 26.749706N | 331 | Extensive | Red soil |
| 65 | 118.487801E | 26.718696N | 436 | Extensive | Red soil |
| 66 | 118.524079E | 26.71071N | 379 | Extensive | Red soil |
| 67 | 118.520656E | 26.712307N | 340 | Extensive | Red soil |
| 68 | 118.507195E | 26.771629N | 366 | Extensive | Red soil |
| 69 | 118.50537E | 26.768206N | 466 | Extensive | Red soil |
| 70 | 118.470233E | 26.810188N | 371 | Extensive | Red soil |
| 71 | 118.470918E | 26.80494N | 427 | Extensive | Red soil |
| 72 | 118.472971E | 26.806765N | 705 | Extensive | Red soil |
| 73 | 118.467951E | 26.806993N | 751 | Extensive | Red soil |
| 74 | 118.434412E | 26.78258N | 610 | Extensive | Red soil |
| 75 | 118.433956E | 26.786687N | 624 | Extensive | Red soil |
| 76 | 118.470461E | 26.741284N | 556 | Extensive | Red soil |
| 77 | 118.472287E | 26.74425N | 782 | Extensive | Red soil |
| 78 | 118.430716E | 26.734876N | 381 | Extensive | Red soil |
| 79 | 118.429575E | 26.733507N | 531 | Extensive | Red soil |
| 80 | 118.410182E | 26.745143N | 505 | Extensive | Red soil |
| 81 | 118.41657E | 26.74925N | 478 | Extensive | Red soil |
| 82 | 118.329873E | 27.103279N | 391 | Extensive | Red soil |
| 83 | 118.361587E | 27.117881N | 116 | Extensive | Red soil |
| 84 | 118.384175E | 27.144119N | 130 | Extensive | Red soil |
| 85 | 118.370714E | 27.207319N | 292 | Extensive | Red soil |
| 86 | 118.38E | 27.167848N | 403 | Extensive | Red soil |
| 87 | 118.345776E | 27.206635N | 198 | Extensive | Red soil |
| 88 | 118.333067E | 27.173552N | 228 | Extensive | Red soil |
| 89 | 118.399233E | 27.19979N | 521 | Extensive | Lateritic red soil |
| 90 | 118.357252E | 27.225115N | 231 | Extensive | Red soil |
| 91 | 118.420224E | 27.187241N | 200 | Extensive | Red soil |
| 92 | 118.4257E | 27.18861N | 398 | Extensive | Red soil |
| 93 | 118.37571E | 27.164197N | 214 | Extensive | Red soil |
| 94 | 118.561006E | 27.312022N | 187 | Extensive | Red soil |
| 95 | 118.525663E | 27.280438N | 794 | Extensive | Red soil |
| 96 | 118.555993E | 27.276428N | 713 | Extensive | Red soil |
| 97 | 118.530425E | 27.256124N | 678 | Extensive | Red soil |
| 98 | 118.472873E | 27.304054N | 651 | Extensive | Red soil |
| 99 | 118.472623E | 27.244898N | 600 | Extensive | Red soil |
| 100 | 118.456981E | 27.265201N | 720 | Extensive | Red soil |
| 101 | 118.462496E | 27.265201N | 604 | Extensive | Red soil |
| 102 | 118.436427E | 27.280241N | 734 | Extensive | Red soil |
| 103 | 118.409456E | 27.253671N | 623 | Extensive | Red soil |
| 104 | 118.499444E | 27.267457N | 692 | Extensive | Red soil |
| 105 | 118.529398E | 27.310571N | 589 | Extensive | Yellowish red soil |
| 106 | 118.501825E | 27.2647N | 514 | Extensive | Yellowish red soil |
| 107 | 118.501699E | 27.247154N | 795 | Extensive | Yellowish red soil |
| 108 | 118.359376E | 27.062195N | 519 | Extensive | Yellowish red soil |
| 109 | 118.294985E | 27.009345N | 136 | Extensive | Red soil |
| 110 | 118.29863E | 27.01299N | 80 | Extensive | Red soil |
| 111 | 118.330522E | 27.026051N | 100 | Extensive | Red soil |
| 112 | 118.80554E | 27.291701N | 138 | Extensive | Red soil |
| 113 | 118.804873E | 27.254637N | 416 | Extensive | Red soil |
| 114 | 118.783502E | 27.24629N | 928 | Extensive | Red soil |
| 115 | 118.725069E | 27.298713N | 244 | Extensive | Red soil |
| 116 | 118.729743E | 27.246624N | 205 | Extensive | Red soil |
| 117 | 118.841936E | 27.204551N | 174 | Extensive | Red soil |
| 118 | 118.79953E | 27.236606N | 649 | Extensive | Red soil |
| 119 | 118.664298E | 27.296042N | 226 | Extensive | Red soil |
| 120 | 118.624563E | 27.261983N | 430 | Extensive | Red soil |
| 121 | 118.731413E | 27.298045N | 324 | Extensive | Red soil |
| 122 | 118.886012E | 27.124414N | 216 | Extensive | Red soil |
| 123 | 118.912658E | 27.149123N | 753 | Extensive | Red soil |
| 124 | 118.850551E | 27.168155N | 656 | Extensive | Red soil |
| 125 | 118.743367E | 27.156135N | 313 | Extensive | Red soil |
| 126 | 118.729343E | 27.176169N | 153 | Extensive | Red soil |
| 127 | 118.768744E | 27.152796N | 169 | Extensive | Red soil |
| 128 | 118.77609E | 27.1164N | 172 | Extensive | Red soil |
| 129 | 118.834857E | 27.134431N | 269 | Extensive | Red soil |
| 130 | 118.852554E | 27.117736N | 765 | Extensive | Yellowish red soil |
| 131 | 118.891288E | 27.083009N | 610 | Extensive | Yellowish red soil |
| 132 | 118.626499E | 27.209894N | 1090 | Extensive | Yellow soil |
| 133 | 118.714317E | 27.147453N | 178 | Extensive | Yellow soil |
| 134 | 118.64019E | 27.108052N | 215 | Extensive | Red soil |
| 135 | 118.712981E | 27.086014N | 176 | Extensive | Red soil |
| 136 | 118.704968E | 27.111391N | 176 | Extensive | Red soil |
| 137 | 118.614145E | 27.197205N | 165 | Extensive | Red soil |
| 138 | 118.676252E | 27.168489N | 271 | Extensive | Red soil |
| 139 | 118.627167E | 27.128421N | 133 | Extensive | Red soil |
| 140 | 118.672913E | 27.123412N | 133 | Extensive | Red soil |
| 141 | 118.635849E | 27.163481N | 147 | Extensive | Red soil |
| 142 | 118.585763E | 27.058968N | 145 | Extensive | Red soil |
| 143 | 118.58977E | 27.060971N | 178 | Extensive | Red soil |
| 144 | 118.594444E | 27.08735N | 198 | Extensive | Red soil |
| 145 | 118.585763E | 27.02324N | 165 | Extensive | Red soil |
| 146 | 118.591105E | 27.026579N | 523 | Extensive | Red soil |
| 147 | 118.645198E | 26.991185N | 428 | Extensive | Red soil |
| 148 | 118.649539E | 26.991185N | 467 | Extensive | Red soil |
| 149 | 118.655215E | 27.019233N | 370 | Extensive | Red soil |
| 150 | 118.655883E | 27.013223N | 481 | Extensive | Red soil |
| 151 | 118.620489E | 27.055963N | 563 | Extensive | Red soil |
| 152 | 118.445522E | 27.126751N | 182 | Extensive | Red soil |
| 153 | 118.451198E | 27.126751N | 296 | Extensive | Red soil |
| 154 | 118.468895E | 27.048951N | 284 | Extensive | Red soil |
| 155 | 118.470565E | 27.043942N | 169 | Extensive | Red soil |
| 156 | 118.483921E | 27.037598N | 126 | Extensive | Red soil |
| 157 | 118.541019E | 27.180844N | 189 | Extensive | Red soil |
| 158 | 118.544692E | 27.177505N | 109 | Extensive | Red soil |
| 159 | 118.518648E | 27.071656N | 178 | Extensive | Red soil |
| 160 | 118.522321E | 27.067983N | 307 | Extensive | Red soil |
| 161 | 118.497611E | 27.098703N | 178 | Extensive | Red soil |
| 162 | 118.002428E | 27.10104N | 251 | Extensive | Red soil |
| 163 | 118.007102E | 27.104379N | 527 | Extensive | Red soil |
| 164 | 118.010441E | 27.071656N | 521 | Extensive | Red soil |
| 165 | 118.016452E | 27.075329N | 556 | Extensive | Red soil |
| 166 | 118.020125E | 27.066648N | 569 | Extensive | Red soil |
| 167 | 118.023464E | 27.07366N | 740 | Extensive | Yellowish red soil |
| 168 | 118.069209E | 27.082341N | 528 | Extensive | Red soil |
| 169 | 118.07188E | 27.085013N | 480 | Extensive | Red soil |
| 170 | 118.130982E | 27.136434N | 459 | Extensive | Red soil |
| 171 | 118.167378E | 27.096032N | 176 | Extensive | Red soil |
| 172 | 118.170049E | 27.103378N | 418 | Extensive | Red soil |
| 173 | 118.176727E | 27.096699N | 285 | Extensive | Red soil |
| 174 | 118.217797E | 27.074995N | 445 | Extensive | Red soil |
| 175 | 118.23683E | 27.052624N | 155 | Extensive | Red soil |
| 176 | 118.245846E | 27.05997N | 218 | Extensive | Red soil |
| 177 | 118.277233E | 27.08134N | 105 | Extensive | Red soil |
| 178 | 118.233491E | 27.13009N | 158 | Extensive | Red soil |
| 179 | 118.233825E | 27.159474N | 130 | Extensive | Red soil |
| 180 | 118.222773E | 27.045946N | 99 | Extensive | Red soil |
| 181 | 118.530208E | 26.93546N | 244 | Extensive | Red soil |
| 182 | 118.537888E | 26.9398N | 606 | Extensive | Red soil |
| 183 | 118.355909E | 27.110093N | 403 | Extensive | Red soil |
| 184 | 118.231362E | 27.12779N | 127 | Extensive | Red soil |
| 185 | 118.203647E | 27.203587N | 129 | Extensive | Red soil |
| 186 | 118.568274E | 27.027952N | 114 | Extensive | Red soil |
| 187 | 118.566938E | 27.023611N | 331 | Extensive | Red soil |
| 188 | 118.573282E | 27.024947N | 430 | Extensive | Red soil |
| 189 | 118.197637E | 27.017267N | 355 | Extensive | Red soil |
| 190 | 118.20131E | 27.018936N | 432 | Extensive | Red soil |
| 191 | 118.19363E | 27.019604N | 472 | Extensive | Red soil |
| 192 | 118.141207E | 27.011924N | 501 | Extensive | Red soil |
| 193 | 118.146549E | 27.06969N | 198 | Extensive | Red soil |
| 194 | 118.139871E | 27.024613N | 367 | Extensive | Red soil |
| 195 | 118.197971E | 26.975862N | 236 | Extensive | Red soil |
| 196 | 118.165916E | 26.989219N | 628 | Extensive | Red soil |
| 197 | 118.135864E | 26.96885N | 234 | Extensive | Red soil |
| 198 | 118.229358E | 26.961504N | 261 | Extensive | Red soil |
| 199 | 118.239709E | 26.981539N | 571 | Extensive | Red soil |
| 200 | 118.227021E | 26.994561N | 601 | Extensive | Red soil |
| 201 | 118.05005E | 27.02528N | 759 | Extensive | Red soil |
| 202 | 118.184281E | 27.040306N | 526 | Extensive | Red soil |
| 203 | 118.083775E | 27.011256N | 580 | Extensive | Red soil |
| 204 | 118.066745E | 27.04231N | 538 | Extensive | Red soil |
| 205 | 118.089117E | 27.03463N | 418 | Extensive | Red soil |
| 206 | 118.103141E | 27.053996N | 336 | Extensive | Red soil |
| 207 | 118.113826E | 27.032292N | 255 | Extensive | Red soil |
| 208 | 118.659819E | 26.880312N | 225 | Extensive | Red soil |
| 209 | 118.127779E | 27.002909N | 274 | Extensive | Red soil |
